# Exogenous Citrulline and Glutamine Contribute to Reverse the Resistance of *Salmonella* to Apramycin

**DOI:** 10.3389/fmicb.2021.759170

**Published:** 2021-10-14

**Authors:** Yan Yong, Yanhong Zhou, Kexin Liu, Guochang Liu, Liqin Wu, Binghu Fang

**Affiliations:** ^1^National Risk Assessment Laboratory for Antimicrobial Resistance of Animal Original Bacteria, South China Agricultural University, Guangzhou, China; ^2^Guangdong Provincial Key Laboratory of Veterinary Pharmaceutics Development and Safety Evaluation, South China Agricultural University, Guangzhou, China; ^3^Guangdong Wens Dahuanong Biotechnology Limited Company, YunFu, China; ^4^Center for Agricultural Product Quality and Safety of Guangdong Province, Guangzhou, China

**Keywords:** citrulline, glutamine, *Salmonella*, apramycin, resistance, reverse

## Abstract

Antibiotic resistance is an increasing concern for human and animal health worldwide. Recently, the concept of reverting bacterial resistance by changing the metabolic state of antibiotic-resistant bacteria has emerged. In this study, we investigated the reversal of Apramycin resistance in *Salmonella*. First, non-targeted metabonomics were used to identify key differential metabolites of drug-resistant bacteria. Then, the reversal effect of exogenous substances was verified *in vivo* and *in vitro*. Finally, the underlying mechanism was studied. The results showed that the metabolites citrulline and glutamine were significantly reduced in Apramycin-resistant *Salmonella*. When citrulline and glutamine were added to the culture medium of drug-resistant *Salmonella*, the killing effect of Apramycin was restored markedly. Mechanistic studies showed that citrulline and glutamine promoted the Tricarboxylic acid cycle, produced more NADH in the bacteria, and increased the proton-motive force, thus promoting Apramycin entry into the bacterial cells, and killing the drug-resistant bacteria. This study provides a useful method to manage infections by antibiotic-resistant bacteria.

## Introduction

Antibiotic-resistant microbes exist widely in human and animal environments and have become a serious public health threat. The inappropriate use of antibiotics has led to the emergence of multi-drug-resistant bacteria (Khameneh et al., 2016; Yelin and Kishony, 2018). Multi-drug-resistant bacteria are a global problem because of their rapid spread and difficulty in treatment. Antibiotic resistance genes exist widely in almost all ecosystems (Larsson, 2014; Nesme and Simonet, 2015). In addition, the antibiotics used in food-producing animals and humans are mostly the same, which increases the risk of the emergence and spread of new drug-resistant bacteria in animals and humans (Van et al., 2012; Maron et al., 2013). Unfortunately, the development of new antibiotics is much slower than that of bacterial resistance (Conly and Johnston, 2005; Escola-Verge et al., 2020).

*Salmonella* is a common foodborne zoonotic pathogen that can be transmitted from animal to animals, animal to humans, and human to animals (Gut et al., 2018). There are more than 2000 *Salmonella* serotypes found in the world. *Salmonella typhi* serotypes are the main cause of human typhoid fever, which is a serious disease in developing countries (Bula-Rudas et al., 2015). Furthermore, *Salmonella* infection causes diarrhea, high fever, and even septicemia, in all kinds of animals at any age (Bao et al., 2019).

Antibiotics are widely used as a growth promotor to stimulate growth of animal from last several decades. Although its application has been prohibited in many countries, early widespread abuse has resulted in emergence of antibiotic resistance microbes. It also increases the risk of the diet-induced transmission of drug-resistant bacteria from animals to humans (Van et al., 2012). The effect of antibiotics on bacterial pathogens, such as *Salmonella typhi*, is declining with time. *Salmonella* has different degrees of resistance to antibiotics in clinical practice (Crump et al., 2015; Wong et al., 2015). In veterinary clinics, the drug resistance of *Salmonella* is more serious (Lebdah et al., 2017; Zhao et al., 2017). In addition, the same *Salmonella* drug resistance genes have been detected in humans and animals, and in animal-derived foods (Fearnley et al., 2011; Ahmed et al., 2014; Voss-Rech et al., 2017). It has been reported that the mechanism of *Salmonella* resistance to a variety of drugs is related to many factors, such as gene mutation, expression of efflux pump, production of inactivating enzyme, movable gene elements, and their transfer (Capuano et al., 2013; Jian et al., 2019). The emergence of drug-resistant bacteria means that new methods are needed to prevent or treat infections caused by intestinal pathogens (Nami et al., 2015).

Aminoglycosides are an important weapon against *Salmonella* infection because of their strong bactericidal effect on Gram-negative bacteria. Aminoglycosides inhibit protein synthesis by binding to the 30S subunit of the bacterial ribosome, thus hindering bacterial reproduction (Wilson, 2014). Apramycin, an aminoglycoside antibiotic for animals, is widely used to treat *Salmonella*, *Escherichia coli*, and other infections (Kang et al., 2017). The most common Apramycin resistance gene is *aac(3)-IV* (encoding aminoglycoside N(3)-acetyltransferase), the transfer of which can lead to the horizontal spread of drug resistance between animals and humans and cross-resistance to Apramycin and gentamicin (Zhang et al., 2009; Michel et al., 2020).

Metabonomics comprises the study of all the metabolites in a cell at a given time. Metabolites can better reflect the cellular environment, which is closely related to the cells’ nutritional status, and the effect of drugs and other external factors. The metabolic status of bacteria significantly affects their antibiotic sensitivity, and specific metabolic characteristics are related to antibiotic resistance (Birkenstock et al., 2012; Antti et al., 2013; Derewacz et al., 2013). Metabonomics can reveal the changes in the intracellular metabolic state of bacteria, identify biomarkers, and provide new strategies against drug resistance (Meylan et al., 2017; Su et al., 2018; Cheng et al., 2019; Li et al., 2020). Various reports showed that the metabolic changes of different bacteria and different drugs vary markedly; therefore, more detailed studies are needed. Previous metabonomic research on drug-resistant bacteria mainly concentrated on human medicine. In veterinary medicine, Only a study on the change of the metabolic state of florfenicol-resistant *Campylobacter* was reported (Li et al., 2015); however, no further research has been performed. The combination of certain substances and drugs could improve the therapeutic effect on drug-resistant bacteria, e.g., thymine combined with ciprofloxacin could kill drug-resistant *Escherichia coli* (Liu et al., 2021); however, how to screen these substances systematically, and determine their mechanism of action, are unclear. In the veterinary field, more research on the metabolic mechanisms of bacterial drug resistance is required, including mechanistic analyses of the reversal mechanism of bacterial drug resistance.

In the present study, we studied the metabonomics of Apramycin-resistant *Salmonella* and sensitive *Salmonella* in order to find out the differential metabolites related to drug resistance. We identified citrulline and glutamine as important metabolites in Apramycin-resistant *Salmonella*, and their reversal effect on resistance was verified using *in vitro* and *in vivo* tests. Citrulline and glutamine could promote the tricarboxylic acid cycle, increase NADH production, and increase the proton-motive force of the cell membrane, ultimately promoting drug entry into the bacterial cells. We propose a new research method and strategy to combat drug-resistant bacteria in animals.

## Materials and Methods

### Chemicals

Apramycin Sulfate was purchased from the China Institute of Veterinary Drug Control (Beijing, China). L-Citrulline and L-Glutamine (Biotechnology grade) were purchased from Shanghai Macklin Biochemical Technology Co., Ltd (Shanghai, China). Mueller Hinton (MH) Agar, MH Broth, Tryptic Soy Agar (TSA), Luria-Bertani (LB) broth, MacConkey Agar, and Xylose Lysine Deoxycholate (XLD) Agar were purchased from Guangdong Huankai Microbial Sci & Tech. Co., Ltd (Guangdong, China). Biotechnology grade cyclophosphamide was purchased from Shanghai Yuanye Bio-Technology Co., Ltd (Shanghai, China). Methanol and acetonitrile (high-performance liquid chromatography grade) were purchased from Thermo Fisher Scientific (Waltham, MA, United States). Carbonyl cyanide m-chlorophenylhydrazone (CCCP), phosphate buffer, and Sodium chloride were purchased from sigma Aldrich (Missouri, United States), GE Healthcare Life Sciences China (Beijing, China), and Shanghai Runjie Chemical Reagent Co., Ltd (Shanghai, China), respectively.

### Bacterial Strains

Standard strains of *Escherichia coli* (ATCC25922) and *Salmonella choleraesuis* (ATCC13312) were purchased from American Type Culture Collection (Manassas, VA, United States), *Salmonella typhimurium* (CICC21484) was purchased from the China Center of Industrial Culture Collection (Beijing, China), and *Salmonella typhi suis* (CVCC3783) was purchased from the China Veterinary Culture Collection Center (Beijing, China). Clinically resistant *Salmonella* strains (SP-JH-Y1407-52, SP-JH-Y1407-31, and SP-JH-Y1407-22) were donated by the Center for Agricultural Product Quality and Safety of Guangdong province (Guangdong, China).

### Induction of Bacterial Antibiotic Resistance

The minimum inhibitory concentrations (MICs) of Apramycin against different *Salmonella* were determined using the microdilution method (Li et al., 2015). The increasing concentration method was used to induce drug-resistant strains. *Salmonella* strains (ATCC13312, CICC21484, and CVCC3783) were inoculated into the broth, and Apramycin at 1/2 MIC concentration was added. The MIC of the bacteria was determined after 1week of continuous culture at 37°C. The culture was continued with the new 1/2 MIC concentration until the MIC of the bacteria increased by more than 32 times, at which point the induction was stopped. The induced drug-resistant strains were passaged in a control medium without any drugs for three generations, and then, the MIC was determined to confirm that stable drug resistance was obtained.

### Metabolomic Analysis

Non-targeted metabonomics studies of Apramycin-resistant *Salmonell*a ATCC13312 (Apr-R-ATCC13312) and a sensitive strain (ATCC13312) were performed. The resistant or sensitive *Salmonella* was cultured in broth to the late logarithmic growth stage. Then, 5ml of the bacterial suspension was taken and centrifuged at 16000g, at −10°C for 10min. The precipitate was washed twice with PBS. The bacteria were collected and quickly quenched in liquid nitrogen to stop their metabolism. Then, 1ml of methanol/acetonitrile/water (2:2:1, V/V) was added, vortexed for 60s, and extracted ultrasonically twice at a low temperature. The supernatant was placed at −20°C for 1h to precipitate proteins. The samples were centrifuged at 20000g at 4°C for 20min, and the supernatant was collected, lyophilized, and stored at −80°C. Before the test, 100μl of acetonitrile/water (1:1, V/V) was added to the lyophilized sample to dissolve the cell metabolites. The samples were separated using ultra-performance liquid chromatography with a HILIC column and analyzed using a triple TOF 5600 mass spectrometer (AB SCIEX, Framingham, MA, United States). Electrospray ionization (ESI) was used to monitor the samples in positive and negative ion modes. To avoid the influence of the fluctuation of the instrument detection signal, continuous analysis of the samples was carried out in random order. Quality Control (QC) samples were inserted into the sample queue to monitor and evaluate the stability of the system to ensure the reliability of the data. The mass spectrometry data were processed by Software Analyst 1.6. The total ion diagram and MRM multi peak diagram of the sample were obtained. According to the retention time and peak type of metabolites, the mass spectrum peaks detected by each metabolite in different samples were corrected again. The original data were converted into MzXML format by Proteo Wizard, and then, the XCMS program was used for peak alignment, retention time correction, and peak area extraction to form a data set for statistical analysis. Multi-dimensional and single-dimensional statistical analysis were performed, including unsupervised principal component analysis (PCA), supervised partial least squares discriminant analysis (PLS-DA), orthogonal partial least squares discriminant analysis (OPLS-DA), T-tests, and multiple of variation analysis.

### Metabolite Supplementation

Different Apramycin-induced resistant *Salmonella* was cultured in broth. In the same bacterial culture medium, different treatments were carried out, including adding the drug alone, adding the exogenous substances alone, and adding the drug and the exogenous substances at the same time. The bacteria of each group were cultured at 37°C for 8h. The cultures were sampled at 0, 2, 4, 6, and 8h for bacterial counting, and bacterial survival curves were drawn. The effects of different concentrations of exogenous substances on resistant bacteria were also investigated. In addition, the same method was used to verify the reversal effect of exogenous substances on clinically isolated resistant bacteria.

### Inhibitory Effect of Carbonyl Cyanide M-Chlorophenylhydrazone on the Reversal of Drug Resistance

Apramycin; two exogenous substances (citrulline plus glutamine); Apramycin plus citrulline and glutamine; and Apramycin plus citrulline and glutamine plus 20μM CCCP were added into the culture medium of Apramycin-induced drug-resistant *Salmonella* (Apr-R-ATCC13312). The bacteria were counted at 0, 2, 4, 6, and 8h, and bacterial survival curves were drawn. The survival rate of each group was calculated in comparison with the bacteria in the culture medium without treatment.

### Measurement of the Membrane Potential

Different exogenous substances were added into the culture medium of Apramycin-resistant *Salmonella* (Apr-R-ATCC13312 and Apr-R-CICC21484), which were then cultured at 37°C for 8h. Then, exactly 1ml of the bacterial solution was removed and centrifuged at 20000g for 5min at −10°C. The supernatant was discarded; the precipitate was washed twice with PBS and then diluted using PBS to approximately 1×10^6^ cells per ml. Then, the membrane potential was measured using a BacLight Bacterial Membrane Potential Kit (Life Technologies, Carlsbad, CA, United States) according to the manufacturer’s instructions.

### NADH Measurements

Three induced drug-resistant *Salmonella* (Apr-R-ATCC13312, Apr-R-CICC21484, and Apr-R-CVCC3783) and a clinical drug-resistant *Salmonella* (SP-JH-Y1407-22) were chosen for the NADH measurement study. 300μl of Apramycin-resistant bacterial culture medium was sampled and added into MH broth with 120mm citrulline and 120mm glutamine, 120mm citrulline only, 120mm glutamine only, and MH broth without any exogenous substances. The bacterial culture of each group was then incubated at 37°C for 8h, at which point exactly 1ml of bacterial culture medium was removed, centrifuged at 20000g for 5min at −10°C, the supernatant was discarded, and the precipitate was washed twice with cold PBS. The bacterial precipitate was placed in a 1.5ml centrifuge tube, extracted, and detected using a NAD ^+^/NADH assay kit (BioAssay Systems, Hayward, CA, United States).

### Determination of the Intracellular Drug Concentration

Apramycin-resistant *Salmonella* was inoculated into 10ml of MH broth containing Apramycin, Apramycin plus citrulline and glutamine, Apramycin plus citrulline, or Apramycin plus glutamine, respectively. After incubation at 37°C for 8h, 10ml of the culture was taken and centrifuged at 20000g for 5min at −10°C. The supernatant was discarded and the precipitate was washed with saline twice. The bacterial cells were added with 0.5ml saline, vortexed for 30s, and then treated with ultrasound for 10min. After centrifugation, the supernatant was collected, and the concentration of Apramycin was determined using an Apramycin ELISA kit (Shanghai Enzyme-linked Biotechnology Co., Ltd., Shanghai, China). The detection limit of the kit was 0.01ng/ml, the accuracy of the method was greater than 80%, the internal plate error of the absorbance of the kit was less than 8%, the inter plate error was less than 15%, and the standard curve ranged from 0.05ng/ml to 4.05ng/ml. This experiment was carried out on three induced drug-resistant strains (Apr-R-ATCC13312, Apr-R-CICC21484, and Apr-R-CVCC3783) and one clinical drug-resistant strain (SP-JH-Y1407-22).

### Validation Using a Mouse Infection Model

Ninety healthy female mice were divided into nine groups, with 10 mice in each group. The groups comprised the negative control group without bacterial infection, the positive control group (mice infected with drug-resistant bacteria but without any treatment), and the other seven groups were treated with Apramycin, citrulline, glutamine, Apramycin plus citrulline, Apramycin plus glutamine, or Apramycin plus citrulline plus glutamine, respectively, after infection with drug-resistant bacteria. All animals were tested and found to be *Salmonella typhimurium*-free before the start of experiments.

Eighty mice were intraperitoneally injected with cyclophosphamide for 5days to establish a neutropenia model. After the establishment of the model, 0.1ml Apramycin-induced *Salmonella typhimurium* (Apr-R-CICC21484) suspension was injected intraperitoneally at a concentration of 10^7^ colony-forming units/ml. At 12h after infection, the mice in the experimental group were injected intraperitoneally with Apramycin or citrulline and glutamine. The doses of Apramycin, citrulline, and glutamine used in the experiment were 20mg/kg body weight (b.w.), 240mg/kg b.w., and 200mg/kg b.w., respectively. At 24h after treatment, all mice were euthanized. The liver, spleen, and blood of all mice were collected under sterile conditions and placed in saline. The tissue or blood of each mouse was homogenized separately, diluted with saline, and inoculated on different nutrient agar plates. Finally, the bacterial load of each mouse liver, spleen, and a blood sample was calculated to evaluate the effect of different treatment schemes.

### Statistical Analysis

Statistical analysis was performed on all the data from the metabolite supplement approach, measurement of the membrane potential, NADH measurements, determination of the intracellular drug concentration, and validation using a mouse infection model experiments using SPSS Statistics 26.0 software (IBM Corp., Armonk, NY, United States).

### Ethics Statement

This study was approved by the Animal Ethics Committee of South China Agricultural University (application NO:2019B192) and was carried out in accordance with the ARRIVE guidelines. The mice in the study were euthanized by cervical dislocation.

## Results

### Resistance Levels of Resistant *Salmonella* Strains

After induction culture with Apramycin, three Apramycin-resistant *Salmonella* strains were obtained. The MIC value of the induced drug-resistant bacteria was up to 40 times higher than that before induction (Table 1). Three clinical isolates of *Salmonella* (SP-JH-Y1407-52, SP-JH-Y1407-31, and SP-JH-Y1407-22) were highly resistant to Apramycin and showed partial resistance to florfenicol, tetracycline, enrofloxacin, and other commonly used veterinary drugs (Table 2).

**Table 1:** MIC values of Apramycin against different *Salmonella* before and after drug induction (μg/ml).

	ATCC13312	CICC21484	CVCC3783
Before drug induction	2	2	1
After drug induction	80	80	40

**Table 2:** MIC values of different drugs on clinical isolates of *Salmonella* (μg/ml).

	SP-JH-Y1407-52	SP-JH-Y1407-31	SP-JH-Y1407-22
Apramycin	256	256	128
Florfenicol	32	32	16
Tetracycline	16	32	16
Gentamycin	8	16	16
Enrofloxacin	2	1	2

### Metabolic Profile and Potential Biomarkers of Apramycin-Resistant *Salmonella*

**Figure 1:**
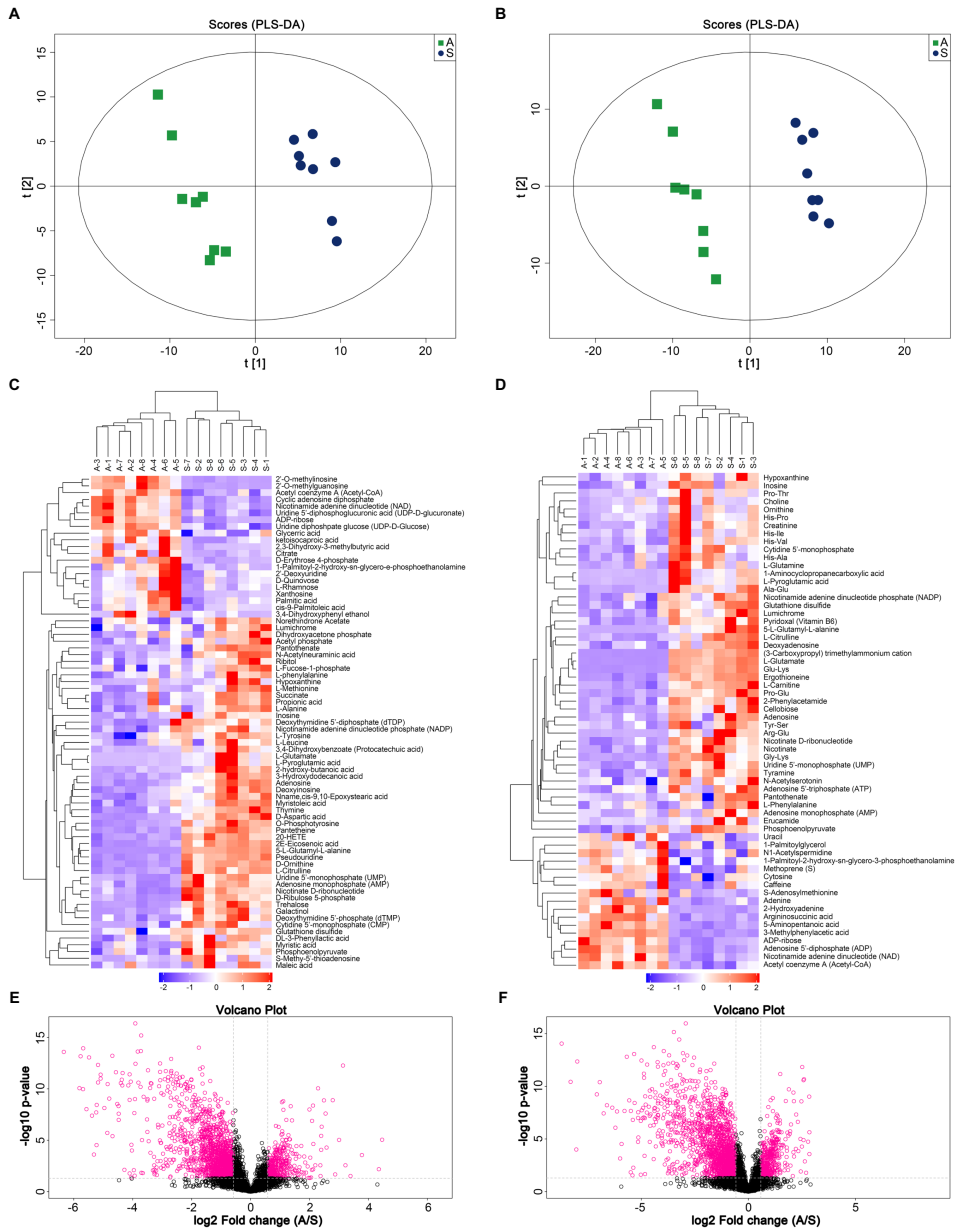
Metabolic profile analysis of Apramycin-resistant and sensitive Salmonella. **(A)** PLS-DA score of Apramycin-resistant and sensitive *Salmonella* in negative ESI modes. **(B)** PLS-DA score of Apramycin-resistant and sensitive *Salmonella* in positive ESI modes. **(C)** Cluster analysis of different metabolites in negative ESI modes. **(D)** Cluster analysis of different metabolites in positive ESI modes. **(E)** Volcano map of the metabolites of Apramycin-resistant *Salmonella* in negative ESI modes. **(F)** Volcano map of the metabolites of Apramycin-resistant Salmonella in positive ESI modes. “A” represents Apramycin-induced resistant *Salmonella* ATCC13312 and “S” represents Apramycin-sensitive *Salmonella* ATCC13312.

A non-targeted metabonomic experiment was conducted in Apramycin-resistant and sensitive *Salmonella*. The experimental bacteria were divided into two groups, with eight biological replicates in each group. The results showed that the response intensity and retention time of the chromatographic peaks of the QC samples overlapped, indicating that the variation caused by the instrument was small during the experiment. We first checked the data integrity and normalized the data between samples and metabolites to ensure that the samples and metabolites could be compared in parallel. The PLS-DA scores of the drug-resistant and sensitive groups are shown in Figures 1A,B. This multi-dimensional statistical analysis showed that the samples in each experimental group were gathered well, while the separation trend of samples between groups was obvious, indicating that the intracellular metabolites of the drug-resistant and sensitive bacteria were quite different.

According to the variable importance for prediction (VIP) analysis obtained using the OPLS-DA model, the influence intensity and explanatory ability of the expression pattern of each metabolite on the classification and discrimination of each group of samples were measured, and the biologically significant differential metabolites were mined. Metabolites with a VIP > 1 and a value of *p* <0.05 were identified as having significant differences. For a more comprehensive and intuitive evaluation of the rationality of candidate metabolites, and to determine the relationship between samples and the differences of metabolite expression patterns in different samples, we used the abundance of significantly different metabolites to cluster the samples. Figures 1C,D shows the hierarchical clustering results of significantly different metabolites under positive and negative ion modes. The same group of samples can appear in the same cluster. Volcano plots (Figures 1E,F) were constructed to analyze and display the changes of metabolites more intuitively. The results showed that 115 metabolites were significantly different between Apramycin-resistant and the sensitive strains, among which 34 increased and 81 decreased. These metabolites were mainly amino acids and their metabolites; products or intermediates of energy metabolism; and substances involved in the tricarboxylic acid cycle, such as citrulline, arginine, glutamine, lysine, Ornithine, ATP, ADP, phosphoenolpyruvate, and succinate.

### Exogenous Citrulline and Glutamine Alter the Sensitivity of Resistant Strains to Apramycin

**Figure 2:**
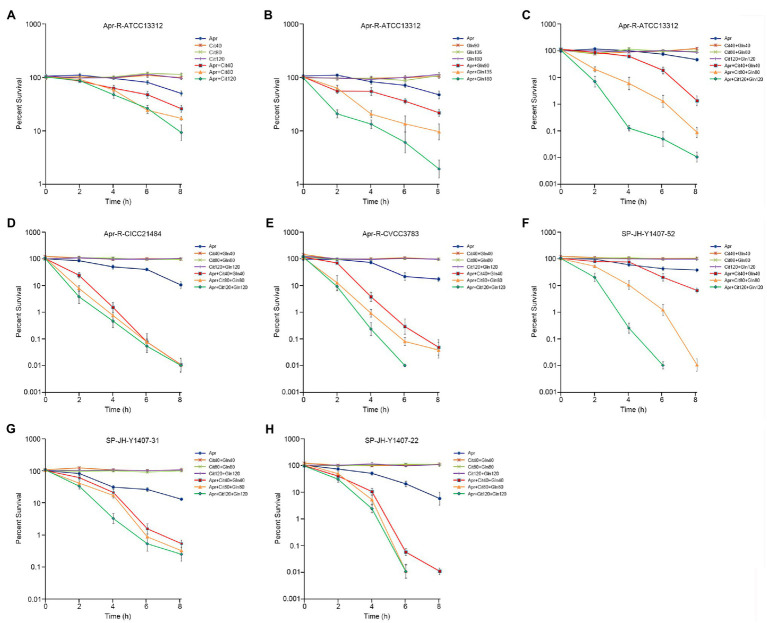
Effect of exogenous citrulline and glutamine on induced and clinical isolates of Apramycin-resistant *Salmonella*. **(A)** Effect of different doses of citrulline on Apramycin-induced resistant *Salmonella* ATCC13312. **(B)** Effect of different doses of glutamine on Apramycin-induced resistant *Salmonella* ATCC13312. **(C)** Effect of citrulline and glutamine on Apramycin-induced resistant *Salmonella* ATCC13312. **(D)** Effect of citrulline and glutamine on Apramycin-induced resistant *Salmonella* CICC21484. **(E)** Effect of citrulline and glutamine on Apramycin-induced resistant *Salmonella* CVCC3783. **(F)** Effect of citrulline and glutamine on clinical isolated drug-resistant *Salmonella* SP-JH-Y1407-52. **(G)** Effect of citrulline and glutamine on clinical isolated drug-resistant *Salmonella* SP-JH-Y1407-31. **(H)** Effect of citrulline and glutamine on clinical isolated drug-resistant *Salmonella* SP-JH-Y1407-22. “Apr” represents Apramycin; “Cit40, Cit80, and Cit120” represent citrulline at a concentration of 40mm, 80mm, and 120mm, respectively; and “Gln90, Gln135, Gln180, Gln40, Gln80, and Gln120” represent glutamine at a concentration of 90mm, 135mm, 180mm, 40mm, 80mm, and 120mm, respectively.

Different concentrations of citrulline and glutamine were added to the culture medium containing a fixed concentration of Apramycin. Viable bacteria were counted at different times. The results showed that citrulline and glutamine promoted Apramycin killing of drug-resistant *Salmonella*, and over a certain range, the concentration of exogenous substances correlated positively with the intensity of the synergistic bactericidal effect. When 120mm citrulline, 120mm glutamine, and 1/2 MIC Apramycin were added, the killing rate of drug-resistant bacteria was 99.99% after 8h. The same effect was observed for the clinical drug-resistant *Salmonella*. The survival rate of drug-resistant *Salmonella* in the test group treated with citrulline, glutamine, and Apramycin was significantly lower than that in the test group treated with Apramycin alone from 2h after culture (*p* <0.05). Thus, citrulline and glutamine could help Apramycin to kill nearly all clinical drug-resistant bacteria after 8h of culture. The details are shown in Figure 2.

### Exogenous Citrulline and Glutamine Promote Drug Entry Into Cells

**Figure 3:**
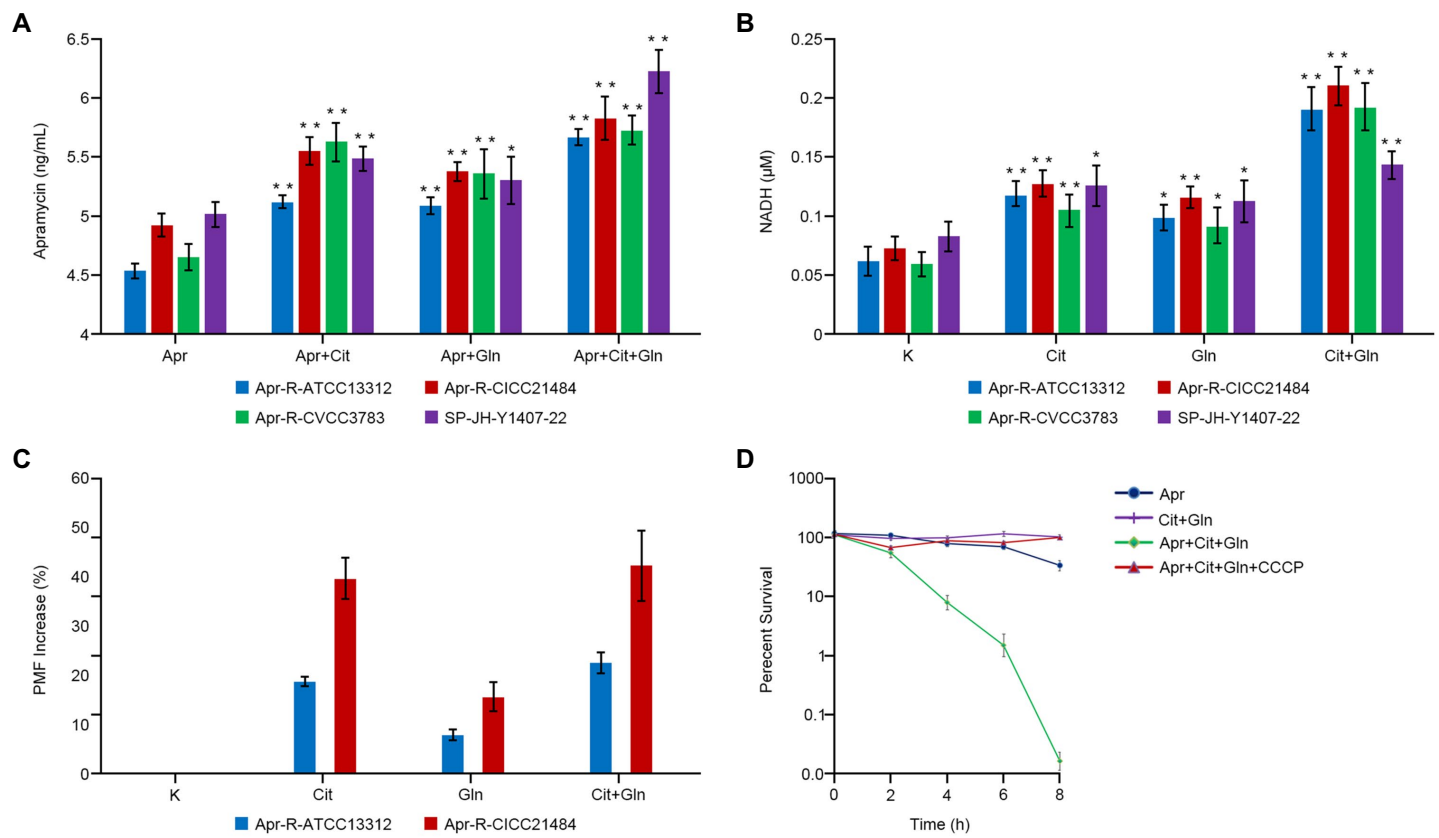
Effect of exogenous citrulline and glutamine on intracellular drugs, NADH, and proton-motive force (PMF). **(A)** Concentration of Apramycin in bacteria cultured with different exogenous substances. **(B)** The NADH content of bacteria cultured with different exogenous substances. **(C)** The PMF of Apramycin-resistant *Salmonella* cultured with citrulline and (or) glutamine. **(D)** Survival curve of bacteria in different media with or without CCCP. “Apr” represents Apramycin alone, “Apr+Cit” represents the combination of Apramycin and citrulline; “Apr+Gln” represents the combination of Apramycin and glutamine; “Apr+Cit+Gln” represents the combination of Apramycin, citrulline, and glutamine; “K” represents the control group; “Cit” represents citrulline alone; “Gln” represents glutamine alone; and “Cit+Gln” represents citrulline plus glutamine. Results are displayed as the mean±SEM, and significant differences are identified (^*^p <0.05; ^**^p <0.01, compared with the group without any exogenous substances) as determined using SPSS Statistics 26.0. Three biological repeats were carried out.

Drug-resistant *Salmonella* was cultured in the medium containing Apramycin, Apramycin plus citrulline, Apramycin plus glutamine, and Apramycin plus citrulline plus glutamine for 8h; then, the concentration of Apramycin in bacterial cells under different culture conditions was determined by an ELISA method. The results showed that when the drug-resistant *Salmonella* was cultured with Apramycin plus citrulline and/or glutamine, the intracellular drug contents of different serotypes of induced drug-resistant *Salmonella* and clinical drug-resistant *Salmonella* were significantly higher than those of the control group treated with Apramycin alone (*p* <0.05). However, when citrulline and glutamine were added at the same time, the intracellular drug concentration was the highest (Figure 3A).

### Exogenous Citrulline and Glutamine Promote the Production of NADH

Citrulline alone, glutamine alone, and the combination of citrulline and glutamine were added to the culture medium of different drug-resistant *Salmonella*. After 8h of culture, the intracellular concentration NADH in the bacteria was detected. The intracellular NADH content of all drug-resistant bacteria increased by more than three times when citrulline and glutamine were added together compared with that in the untreated control group (*p* <0.01). The NADH content in the resistant bacteria cultured with either citrulline or glutamine alone was also higher than that in the control group (Figure 3B).

### Exogenous Substances Increase the Proton-Motive Force of Drug-Resistant Bacteria

Different exogenous substances were added to the culture medium of Apramycin-resistant *Salmonella*, which was then cultured at 37°C for 8h. The membrane potential of bacteria in the different experimental groups increased significantly with the addition of citrulline or glutamine. Moreover, the membrane potential of two Apramycin-resistant *Salmonella* (Apr-R-ATCC13312 and Apr-R-CICC21484) cultured with both citrulline and glutamine increased by 22.7 and 42.2%, respectively, compared with that in the untreated control group. Next, citrulline, glutamine, CCCP, (a chemical inhibitor of oxidative phosphorylation), and Apramycin were added to the culture medium of drug-resistant *Salmonella* at the same time, and the bacteria in the culture medium were counted at different times. The results showed that the drug-resistant bacteria grew normally, i.e., in the presence of CCCP, the exogenous substances lost their ability to reverse drug resistance (Figures 3C,D).

### Verification of the Reversal Effect in a Mouse Infection Model

**Figure 4:**
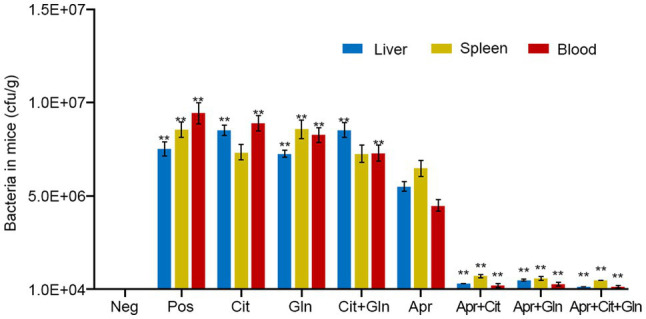
Effects of exogenous citrulline and glutamine combined with Apramycin in the mice infection model. “Pos” represents positive control group; “Cit” represents mice treated with citrulline alone, “Gln” represents mice treated with glutamine alone, “Cit+Gln” represents mice treated with citrulline and glutamine, “Apr” represents mice treated with Apramycin alone, “Apr+Cit” represents mice treated with Apramycin and citrulline, “Apr+Gln” represents mice treated with Apramycin and glutamine, and “Apr+Cit+Gln” represents mice treated with Apramycin, citrulline, and glutamine. Results are displayed as the mean±SEM, and significant differences are identified (^**^p <0.01, compared with the mice treatment with Apramycin alone) as determined using SPSS Statistics 26.0.

Mice were injected intraperitoneally with Apramycin-resistant *Salmonella typhimurium* (Apr-R-CICC21484) to establish a mouse model of drug-resistant bacterial infection. The infected mice showed depression, diarrhea, and death, and the Apramycin-resistant CICC21484 strain was isolated from the liver, spleen, and blood of the infected mice. The infected mice were treated with Apramycin and different exogenous substances, and the results showed that citrulline (240mg/kg b.w) and glutamine (200mg/kg b.w) combined with Apramycin (20mg/kg b.w) significantly improved the body condition of the infected mice. In addition, the bacterial levels in the liver, spleen, and blood of the combined treatment group were significantly lower than in those treated with Apramycin alone (*p* <0.01). Moreover, single citrulline or glutamine combined with Apramycin treatment also showed a good therapeutic effect, and the number of bacteria in the mice was significantly lower than that in mice treated with Apramycin alone (*p* <0.01; Figure 4). Thus, citrulline and glutamine combined with Apramycin could be used to treat an infection with Apramycin-resistant *Salmonella*.

## Discussion

Studies have shown that the metabolic pathways of alanine, aspartate, and glutamate of kanamycin-resistant *Edwardsiella* tarda were altered significantly compared with those of the sensitive strain (Peng et al., 2015). Studies on drug-resistant *Vibrio alginolyticus* showed that the metabolic status of the bacteria was different in the context of different drug resistances. The pyruvate metabolism of ceftazidime-resistant bacteria was decreased, and fatty acid synthesis was increased. During levofloxacin resistance, the metabolism of tricarboxylic acid cycle was weakened, and the pyruvate cycle was also decreased after gentamicin resistance (Liu et al., 2019; Zhang et al., 2019). Similarly, many reports have shown that a high abundance of endogenous metabolites, such as indole, nitric oxide, hydrogen sulfide, and gaseous ammonia, was associated with increased bacterial resistance to antibiotics (Gusarov et al., 2009; Lee et al., 2010; Shatalin et al., 2011; Vitko et al., 2015; Cohen et al., 2016). These studies revealed that the metabolic state of bacteria changes when drug resistance occurs.

In this study, we conducted a non-targeted metabonomic study on Apramycin-resistant and sensitive *Salmonella*. The levels of 81 metabolites, including citrulline, glutamine, succinic acid, leucine, and ATP, decreased significantly in Apramycin-resistant *Salmonella*, while the contents of 34 metabolites, including nicotinamide adenine dinucleotide, adenosine 5ʹ-diphosphate, and acetyl coenzyme A, increased significantly. Enrichment analysis identified the main metabolic pathways as alanine, aspartate, and glutamate metabolism; citrate cycle; glutathione metabolism; arginine biosynthesis; and ABC transporters. We further confirmed that the bacteria’s metabolic state changed markedly after drug resistance. Some of the altered metabolic pathways identified in this study are the same as those reported for kanamycin-resistant *Edwardsiella* tarda (e.g., alanine, aspartate, and glucose metabolism; arginine and proline metabolism; and aminoacyl tRNA biosynthesis) These metabolic pathways might be common in aminoglycoside-resistant bacteria.

To study the reversal effect *in vitro*, experiments were carried using different serotypes of Apramycin-resistant *Salmonella*. The addition of citrulline or glutamine helped to restore the killing effect of Apramycin in these Apramycin-resistant *Salmonella*, and their combination had a more significant effect. This suggested that citrulline and glutamine could restore the effect of Apramycin on drug-resistant *Salmonella* without serum selectivity. The reversal effect was verified using three clinical isolates of drug-resistant *Salmonella*. Clinically, it is difficult to obtain *Salmonella* that is only resistant to Apramycin, indeed the three clinical drug-resistant strains were also resistant to enrofloxacin, florfenicol, gentamicin, and doxycycline. Thus, citrulline and glutamine, as exogenous additives, can reverse the resistance of multi-drug-resistant *Salmonella* to Apramycin, providing a powerful weapon to treat multi-drug-resistant *Salmonella* infection. We established a model of mouse infection with Apramycin-resistant *Salmonella typhimurium*. The effects of different exogenous substances and drugs were assessed in symptomatic mice using the number of bacteria in the mice as the evaluation index. When citrulline and glutamine were added together, Apramycin had the most significant killing effect on drug-resistant *Salmonella* in mice, which was consistent with the *in vitro* results, indicating that this study has important clinical application value.

The mechanism by which the exogenous substances recovered the sensitivity to Apramycin in drug-resistant bacteria was investigated. First, to exert its bactericidal role, Apramycin must enter the bacterial cells. We observed that adding citrulline and glutamine to the culture medium increased the Apramycin content in the drug-resistant bacteria cells significantly compared with that in the untreated control group.

The addition of exogenous glucose and alanine to the culture medium of kanamycin-resistant bacteria increased the membrane proton-motive force of the bacterial cell membrane, thereby promoting drug entry into the bacteria (Su et al., 2015). In this study, the membrane potential of drug-resistant *Salmonella* incubated with exogenous substances, especially their combination, was significantly higher than that without any exogenous substances. NADH is an important electron and proton donor in organisms. Therefore, the NADH content in bacterial cells after adding the exogenous substances was determined, which showed that the NADH content in drug-resistant bacteria treated with citrulline and glutamine increased significantly. Increased NADH would enhance the proton-motive force of drug-resistant *Salmonella*, thus promoting Apramycin entry into the cells to exert its bactericidal effects. Furthermore, in drug-resistant bacteria treated with the exogenous substances, the electron transport chain inhibitor CCCP, and Apramycin, Apramycin could not kill the drug-resistant bacteria, i.e., CCCP abrogated the reversal effect of the exogenous substances. This suggested that the exogenous substances promoted Apramycin efficacy *via* oxidative phosphorylation.

**Figure 5:**
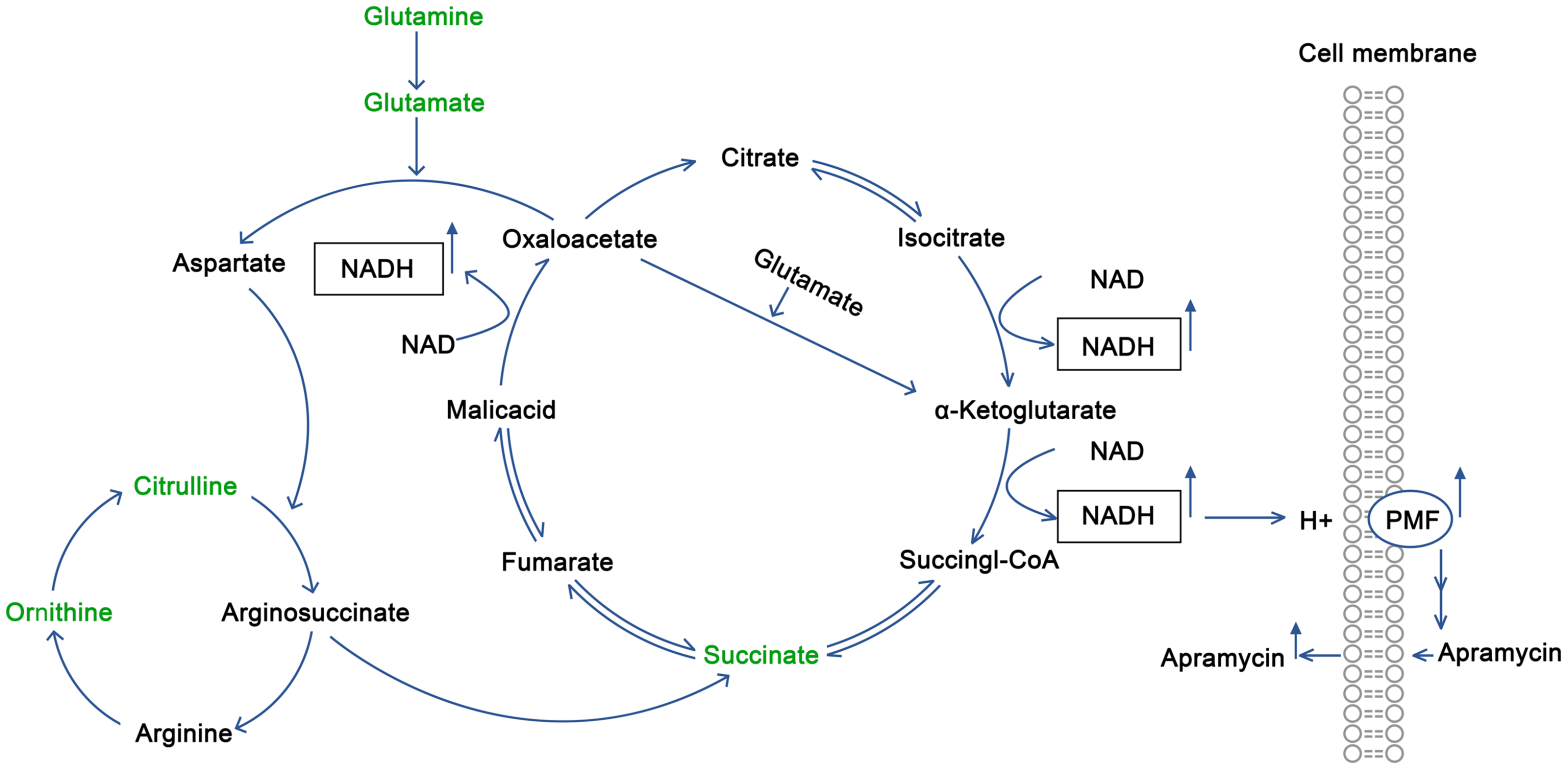
Mechanism by which exogenous citrulline and glutamine promote the recovery of sensitivity of drug-resistant *Salmonella* to Apramycin.

Citrulline is involved in the synthesis of arginine *in vivo*. It condenses with aspartate to form argininosuccinate, which is then cleaved to arginine and fumarate. Fumarate enters the tricarboxylic acid cycle to form oxaloacetate, accompanied by the production of NADH. Oxaloacetate and glutamate then produce aspartate, under the action of glutamic oxaloacetic acid transaminase, and continue to react with citrulline to produce argininosuccinate, thus forming a virtuous cycle that constantly produces NADH. Glutamine is used to produce glutamate *via* deamination. Glutamate and oxaloacetate are used to produce aspartate and α-ketoglutarate, and aspartate enters into the above metabolic process. Alternatively, α-ketoglutarate can enter into the tricarboxylic acid cycle and produce succinyl CoA and NADH at the same time. Thus, citrulline and glutamine promote the tricarboxylic acid cycle and promote the production of NADH, thus increasing the proton-motive force of the bacterial membrane, and finally promote drug entry into cells to kill bacteria. The schematic diagram is shown in Figure 5.

In this study, metabonomics was used to analyze the metabolic changes in drug-resistant bacteria and to identify the key metabolites. The reversal effects of different metabolites on antibiotic resistance were verified *in vitro* and *in vivo*. The reversal mechanism of citrulline and glutamine comprised promotion of metabolism *via* the tricarboxylic acid cycle, increasing the proton-motive force of drug-resistant bacteria, and promoting the entry of the drug into bacteria to achieve a bacterial killing effect. This study provided a powerful weapon for the clinical treatment of drug-resistant bacterial infection and a reference for drug resistance research.

## Data Availability Statement

The original contributions presented in the study are included in the article/supplementary material, and further inquiries can be directed to the corresponding author.

## Ethics Statement

The animal study was reviewed and approved by the Animal Ethics Committee of South China Agricultural University.

## Author Contributions

YY carried out the main experiments and data analysis and wrote the manuscript. YZ and KL participated in the *in vitro* and *in vivo* validation tests. GL took part in animal study. LW participated in the isolation and identification of clinical drug-resistant bacteria. BF conceived and designed the experiments. All authors contributed to the article and approved the submitted version.

## Funding

This work was funded by the Local Innovative and Research Teams Project of Guangdong Pearl River Talents Program (no. 2019BT02N054).

## Conflict of Interest

GL is employed by the Guangdong Wens Dahuanong Biotechnology Limited Company.

The remaining authors declare that the research was conducted in the absence of any commercial or financial relationships that could be construed as a potential conflict of interest.

## Publisher’s Note

All claims expressed in this article are solely those of the authors and do not necessarily represent those of their affiliated organizations, or those of the publisher, the editors and the reviewers. Any product that may be evaluated in this article, or claim that may be made by its manufacturer, is not guaranteed or endorsed by the publisher.
